# Smoking before the birth of a first child is not associated with increased risk of breast cancer: findings from the British Women's Heart and Health Cohort Study and a meta-analysis

**DOI:** 10.1038/sj.bjc.6601916

**Published:** 2004-06-29

**Authors:** D A Lawlor, S Ebrahim, G Davey Smith

**Affiliations:** 1 Department of Social Medicine, University of Bristol, Canynge Hall, Whiteladies Rd, Bristol BS7 8QA, UK

**Keywords:** smoking, breast cancer, first pregnancy, epidemiology

## Abstract

It has been suggested that the period between puberty and first birth is a time when the breast is particularly susceptible to carcinogenic effects. In a cohort of 3047 women aged 60–79 years (*N*=139 breast cancer cases), we found no association between smoking before the birth of a first child and breast cancer risk: fully adjusted (for age, number of children, age at birth of first child, age at menarche, age at menopausal, hysterectomy and/or oophorectomy, ever use of oral contraception, use of hormone replacement therapy, alcohol consumption, body mass index, childhood and adulthood social class) odds ratio 1.06 (95% confidence interval: 0.72, 1.56). The pooled estimate from a meta-analysis of our study and 11 previously published studies (*N*=6528 cases) was 1.07 (0.94, 1.22). We conclude that smoking prior to the birth of a first child is not associated with increased risk of breast cancer.

It has been suggested that smoking may play differing roles in the aetiology of breast cancer depending upon the period in a woman's life during which she smokes (Terry and Rohan, 2002). Breast tissue may be most susceptible to environmental carcinogens during rapid cell proliferation and before complete cellular differentiation, that is, between puberty and completion of a woman's first pregnancy. This suggestion is supported by the fact that older age at menarche and early age at first pregnancy, both of which will decrease the length of this susceptible period, are associated with reduced risk of breast cancer (MacMahon *et al*, 1970; Hsieh *et al*, 1990).

A small number of studies that have assessed the association of smoking prior to completion of first pregnancy with breast cancer and have reported conflicting results (Terry and Rohan, 2002). We aimed to assess this association in a large cohort study and in a meta-analysis of all previously published studies.

## MATERIALS AND METHODS

### British Women's Heart and Health Cohort Study

Data from the baseline assessment and first 3 years of follow-up of the British Women's Heart and Health study were used. Full details of baseline assessment have been reported previously (Lawlor *et al*, 2003a, 2003b). In brief, 4286 women were assessed (medical record review, research nurse interview, self-completed questionnaire and physical examination) between 1999 and 2001. These women have been followed up over a median of 3.5 years by flagging with the NHS central register for mortality and cancer register data, two yearly review of their primary-care medical records (in Britain primary-care records contain details of secondary-care treatment) and a recently mailed 3-year follow-up health questionnaire (Q3) sent to all surviving participants between March and September 2003. Of the 4108 survivors, 3704 (90%) responded to this questionnaire. Ethics committee approval was obtained for this study.

### Breast cancer cases

In this paper, we have included all cases of breast cancer reported at baseline assessment (prevalent cases) and those occurring over the median 3.5 years follow-up period (incident cases). Three main sources were used to determine breast cancer status: (i) self-report in the baseline questionnaire and Q3; (ii) a diagnosis recorded in the medical records and (iii) all participants were flagged with the National Health Service central register (NHSCR) that provided details of cancer registrations. Anyone with a diagnosis of breast cancer from any one of these three sources was considered to be a case. Five additional incident cases that had not been identified by any of these three sources were obtained because they occurred as the underlying cause or elsewhere on the woman's death certificate. Thus, this study is of prevalent and incident breast cancer cases occurring any time in the woman's life up to January 2004.

### Assessment of smoking and potential covariates

Age at menopause, age at menarche, number of pregnancies and live births, current and past use of hormone replacement therapy (HRT) and past use of oral contraception, history of a hysterectomy and/or oophorectomy, alcohol and smoking history were obtained from the self-completed questionnaire and/or the research nurse interview (Lawlor *et al*, 2003a, 2003b). Detailed smoking histories included the age at which smoking started. Adult social class was defined on the basis of the longest held occupation of her husband for married women and her own longest held occupation for single women, childhood social class was defined on the basis of the longest held occupation of the participants father, and both were classified according to the Registrar General's classification (Office of Population Censuses and Surveys, 1980). Standing height was measured without shoes using a Harpenden Stadiometer, which recorded to the nearest millimetre. Weight was measured in light clothing without shoes to the nearest 0.1 kg using Soenhle portable scales.

### Statistical analysis

Multiple logistic regression was used to assess the association between smoking in relation to first birth and breast cancer with adjustment for potential confounding factors: age (entered as a continuous variable), age at first birth (indicator: <20, 21–24, 25–29, 30–34, ⩾35), age at menarche (indicator: <11, 11, 12, 13, 14, ⩾15), number of births (indicator: 1, 2, 3, 4, 5, 6+), age at menopause (indicator: <40, 41–44, 45–49, 50, 51, older than 51), use of HRT (ever or never), hysterectomy and/or oophotectomy (binary), childhood and adult social class (indicator: I, II, IIInonmanual, IIImanual, IV, V, unemployed), body mass index (BMI) (continuous) and alcohol consumption (indicator: daily, weekends only, once or twice a month, special occasions, never). To further explore whether smoking around the time of breast development was a risk factor for future breast cancer, we assessed the association of smoking during the period 1 year before menarche and the first 5 years after menarche in all women using multiple logistic regression. For all analyses robust standard errors, which take into account possible nonindependence between women from the same town, were used to calculate confidence intervals (CI) and *P*-values.

### Meta-analysis

Searches of Medline and Embase (up to January 2004) were undertaken using extended terms for breast neoplasia and smoking. Any published study that assessed the association between smoking before a first pregnancy and breast cancer risk was included in a meta-analysis. We decided *a priori* to pool estimates using DerSimonian and Laird's random effects methods, since it was likely that there would be heterogeneity between studies due to differences in the number of pre- and postmenopausal cases and differences in study design (DerSimonian and Laird, 1986). Metaregression analysis was used to assess the effect of menopausal status on heterogeneity between studies (Sterne *et al*, 2001). A series of sensitivity analyses were undertaken in which: (i) two studies with in-pregnancy smoking assessment only were excluded; (ii) one study with smoking only before pregnancy was excluded; (iii) for studies that provided estimates of both any smoking before pregnancy and only smoking before pregnancy, the latter estimates were substituted into the main analysis; (iv) for studies with varying durations of smoking before pregnancy these were substituted into the main analysis starting first with shortest duration. We examined funnel plots and used Egger and Begg tests to determine the extent of small study bias. (Sterne *et al*, 2001) All statistical analyses were undertaken using Stata version 8.

## RESULTS

Of the 4286 women in this study, 225 (5.3%) had breast cancer ascertained from at least one source. The majority (82%) of these cases were identified from at least two sources. The age distribution of women with cancer identified by each source were similar – mean (standard deviation) age of women with breast cancer identified by self report 68.7 (5.3), identified by medical record review 68.9 (5.3) and identified by cancer register 69.0 (5.4). Of the 225 cases, 170 were prevalent and 55 incident cases.

Table 1

**Table 1:** Smoking and other characteristics irrespective of whether women had breast cancer or not (*N*=4286)

	**Percent (95% CI)**		
	**Breast cancer *N*=225**	**No breast cancer *N*=4061**	****P-******value**
Ever smoked	43.1 (36.5, 49.9)	44.8 (43.2, 46.3)	0.63
Current smoker (at baseline assessment)	11.6 (7.7, 16.5)	12.0 (11.0, 13.1)	0.84
Smoked prior to first birth^a^	32.1 (24.5, 40.6)	30.5 (28.9, 32.2)	0.68
Smoked either 1 year before or within 5 years after menarche^b^	25.6 (19.2, 32.8)	26.3 (24.7, 27.9)	0.84
Nulliparous	13.8 (9.4, 19.3)	9.5 (8.6, 10.5)	0.04
Young age and menarche (<11 years)	4.9 (2.5. 8.6)	2.1 (1.7, 2.6)	0.008
Old age at menopause (>51 years)	33.8 (27.6, 40.04)	27.2 (25.9, 28.6)	0.03
Hysterectomy and/or oophorectomy	24.4 (19.0, 30.6)	30.2 (28.8, 31.6)	0.06
Ever used HRT	18.7 (13.8, 24.4)	12.5 (11.5, 13.6)	0.007
Ever used oral contraception	25.4 (19.7, 31.7)	23.9 (22.2, 24.9)	0.53
Childhood manual social class	81.3 (75.6, 86.2)	80.0 (78.7, 81.1)	0.61
Adult manual social class	55.1 (48.4, 61.7)	57.4 (55.9, 58.9)	0.49
Over weight (BMI >25)	71.4 (64.8, 77.1)	67.9 (66.4, 69.4)	0.30
Daily alcohol consumption	21.0 (15.8, 27.1)	18.1 (16.9, 19.4)	0.29
Tee-total	17.8 (12.9, 23.5)	16.5 (15.3, 17.7)	0.63

BMI=body mass index; HRT=hormone replacement therapy; CI=confidence interval.

a Only among those with at least one child and compared to never smokers=139 cases (see text).

b Irrespective of whether they had children or not and compared to never smokers.

shows the distributions of established breast cancer risk factors and other potential risk factors by breast cancer status among all 4286 women. For established risk factors, our results are in the same direction and of similar magnitudes to what one would expect based on consistent results from previous studies. None of the smoking alcohol or social class variables were associated with breast cancer.

### Smoking prior to first birth

Of the 4286 women, 3467 (81%) had at least one live birth and of these 3047 (88%) gave their age at the time that their first child was born. The associations presented in Table 1 were similar in this subgroup of 3047 women. Of these 3047 women, 139 (4.6%) had breast cancer. Table 2

**Table 2:** Breast cancer and other characteristics by smoking in relation to birth of first child among women with at least one live birth and who provided age at first birth (*N*=3047)

	**Percent (95% CI)**			
	**Never smoked *N*=1739**	**Smoked before first birth *N*=933**	**Smoked but only after first birth *N*=376**	***P*-value**
Breast cancer	4.7 (3.8, 5.8)	4.8 (3.6, 6.4)	3.7 (2.2, 6.2)	0.66
Young age and menarche (<11 years)	2.2 (1.6, 3.0)	2.1 (1.4, 3.3)	2.1 (1.1, 4.2)	0.99
Old age at menopause (>51 years)	32.1 (29.9, 34.3)	28.4 (25.6, 31.4)	22.1 (18.2, 26.5)	<0.001
Hysterectomy and/or oophorectomy	30.9 (28.8, 33.2)	29.3 (26.4, 32.3)	34.6 (29.9, 39.5)	0.17
Ever used HRT	13.4 (11.9, 15.1)	16.6 (14.4, 19.1)	12.8 (9.8, 16.5)	0.05
Ever used oral contraception	23.9 (21.9, 26.0)	31.4 (28.5, 34.5)	28.4 (23.8, 33.4)	<0.001
Childhood manual social class	78.9 (76.9, 80.8)	79.4 (76.7, 81.9)	84.3 (80.3, 87.6)	0.05
Adult manual social class	55.4 (53.0, 57.7)	55.3 (52.1, 58.5)	65.4 (60.5, 70.1)	0.001
Over weight (BMI ⩾25)	69.7 (67.4, 71.8)	66.2 (63.0, 69.3)	73.9 (68.9, 78.3)	0.03

BMI=body mass index; HRT=hormone replacement therapy; CI=confidence interval.

shows breast cancer occurrence and the distributions of potential confounding factors by smoking in relation to first birth. There was no association between breast cancer and smoking in relation to first birth. The age-adjusted odds ratio for smoking before the first birth was 1.04 (95% CI: 0.71, 1.51) and the fully adjusted (age, number of children, menarcheal and menopausal age, hysterectomy, use of hormone replacement, use of the oral contraceptive pill, childhood and adulthood social class and BMI) odds ratio was 1.06 (0.72, 1.56). When the analyses were restricted to those who only smoked before their first pregnancy (ie excluding from the analyses all women who smoked but only began after their first birth), the association was essentially unchanged: fully adjusted odds ratio 1.04 (0.67, 1.59). The age-adjusted odds ratios for women who smoked, but only after the birth of their first child compared to never smokers, was 0.79 (0.44, 1.41) and the fully adjusted odds ratio for this association was 0.70 (0.36, 1.36).

### Smoking around the time of puberty

Among all women in the cohort (4286 women with 225 cases of breast cancer), the age-adjusted odds ratio for smoking either in the year prior to or within 5 years of menarche compared to never smoking was 0.96. (0.69, 1.36); the fully adjusted odds ratio was 1.00 (0.70, 1.39).

### Sensitivity analyses

In the whole cohort, 186 (83%) of the total 225 cases were postmenopausal and in the subgroup of women with at least one birth and a known age at first birth 118 (85%) of the 139 breast cancers were postmenopausal. We were therefore unable to determine with any level of precision the effect of associations on premenopausal cancers. The associations for postmenopausal breast cancers only did not differ from those presented and the point estimates for premenopausal cancers were similar to those for postmenopausal cancers and close to the null value (point estimate for premenopausal cancers associated with smoking prior to first birth=0.96, and for smoking around the time of puberty=0.94). When the analyses were restricted to incident cases only, although considerably less precise, the results did not differ from those presented: fully adjusted odds ratio 1.08 (0.39, 2.55). When all analyses were repeated using breast cancer data from each of just one of the three sources (self-report, medical records, cancer register), the results were unchanged.

### Meta-analysis

We identified 11 studies in 10 publications; these are summarised in Table 3

**Table 3:** Summary of studies assessing the association of smoking prior to the birth of a first baby and breast cancer risk

**Study**	**Study type**	**Age at ascertainment/menopausal status**	**Number of cases**	**Number of controls**	**Comparisons**	**OR (95% CI)**	**Adjusted/matched for**
Adami *et al* (1988)	PCC	<45 years over 90% premenopausal	363	454	1–4 years before 1st pregnancy *vs* never	1.0 (0.6, 1.6)	Age, education, age at menarche, age at pregnancy, menopause, benign disease, family history, oral contraceptive use, alcohol consumption
					5–9 years before *vs* never	0.7 (0.4, 1.1)	
					10+ years before *vs* never	0.7 (0.3, 1.4)	
					Any smoking before *vs* never^a^	0.81 (0.59, 1.12)^a^	
Hunter *et al* (1997)	NCC	Mean age 57.6 60% postmenopausal	417	412	1–5 years before 1st pregnancy *vs* never	1.9 (1.2, 2.8)	Year of birth, menopausal status, HRT, age menarche, age first birth, family history, benign disease
					5+ years before *vs* never	1.1 (0.8, 1.6)	
					Any smoking before *vs* never^a^	1.36 (0.84, 2.43)^a^	
Lash *et al* (1999)	PCC	90% postmenopausal	44	145	Only smoked before *vs* never	5.6 (1.5, 21)	Age, history of radiation therapy, BMI, family history, parity, benign disease
Innes *et al* (2001)	PCC	26–45 100% premenopausal	319	768	Smoking during 1st pregnancy *vs* not smoking during 1st pregnancy	3.0 (1.3, 7.2)	Age, maternal age, race and education
Egan *et al* (2002)	PCh f-u 14 years only includes incident cases	Over 60% postmenopausal	1288	70 663	Any smoking before *vs* never	1.12 (0.96, 1.31)^b^	Age, age at menarche, age at first birth, benign disease, family history, menopausal status, age at menopause, weight at age 18 and adult change in weight, adult height, alcohol, carotenoid intake, HRT use
					Only smoked before *vs* never	1.18 (0.95, 1.46)^b^	
Band *et al*, – postmenopausal 2002	PCC	100% postmenopausal	267	267	Any smoking before *vs* never	0.97 (0.76, 1.24)	Age, ethnic origin, marital status, education, alcohol consumption, age at menarche, age at menopause, family history, benign disease, weight and BMI from age 18 years to concurrent, oral contraceptive use, HRT use, reproductive history, breast feeding
Band *et al* (2002)– premenopausal	PCC	100% premenopausal	113	105	Any smoking before *vs* never	1.37 (0.93, 2.01)	As above
Lash *et al* (2002)	PCC	All ages, proportion pre- and postmenopausal not stated	276	258	Any smoking before *vs* never active or passive	0.69 (0.49, 0.96	Age, radiation therapy, BMI, family history, benign disease, alcohol, parity
					Only smoking before *vs* never active or passive	0.73 (0.42, 1.30)	
Kropp *et al* (2002)	PCC	⩽50 77% premenopausal					
			196	469	Any smoking before *vs* never active or passive	1.32 (0.86, 2.03)	Age, study region, alc0hol, breast feeding, education, family history, menopausal status, BMI
					Only smoking before *vs* never active or passive	0.92 (0.52, 1.65)	
Fink *et al* (2003)	PCC	25–55 menopausal status not given	1665	4972	Smoking during pregnancy *vs* not smoking during pregnancy	0.97 (0.80, 1.2)	Age, education, race, parity, history of terminations.
Reynolds *et al* (2004)	PCh 5 years f-u only includes incident cases	75% postmenopausal at baseline	1441	Approx 100 000	<5 years before 1st pregnancy *vs* never	0.99 (0.80, 1.21)^b^	Age, race, family history, age menarche, age first pregnancy, physical activity, alcohol, BMI, menopausal status, BMI × menopause interaction, HRT
					⩾5 years before *vs* never	1.13 (1.00, 1.28)^b^	
					Any smoking before *vs* never^a^	1.08 (0.96, 1.22)^a^	
Lawlor *et al* (2004, this study)	PCh 3 years f-u includes incident and prevalent cases	60–82 85% postmenopausal	139	2908	Ever smoking before *vs* never	1.06 (0.72, 1.56)	Age, age at first birth, number of children, age menarche, age menopause, hysterectomy and/or oophorectomy, ever use of oral contraception, use of hormone replacement therapy, alcohol consumption, BMI, childhood and adulthood social class
					Only smoking before *vs* never	1.04 (0.68, 1.57)	

OR: odds ratio; CI: confidence interval; NCC: nested case–control – this study was nested in a population, prospective cohort; PCC: population-based case–control; PCh: population cohort; HRT: hormone replacement therapy, BMI: body mass index; f-u: follow-up.

a Not provided in the paper but calculated from the data provided – these values used in meta-analysis.

b Hazard ratios.

**Figure 1:**
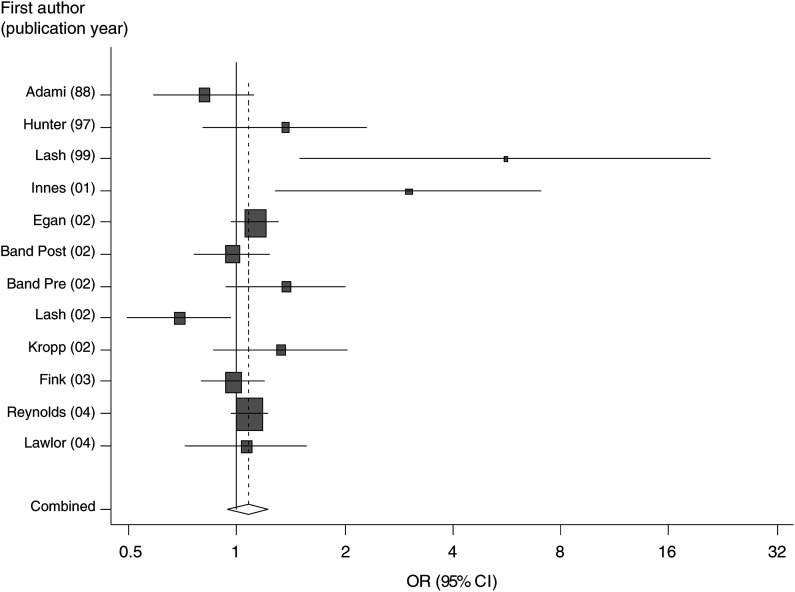
Meta-analysis of studies assessing the effect of smoking before/during first pregnancy with breast cancer risk.

**Figure 2:**
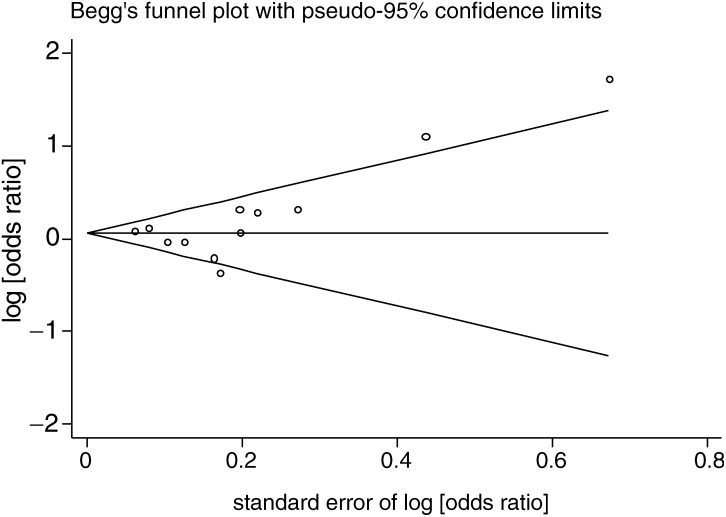
Funnel plot of studies of association of smoking prior to first birth and breast cancer risk.

. (Adami *et al*, 1988; Hunter *et al*, 1997; Lash and Aschengrau, 1999; Innes and Byers, 2001; Egan *et al*, 2002; Band *et al*, 2002; Lash and Aschengrau, 2002; Kropp and Chang-Claude, 2002; Fink and Lash, 2003; Reynolds *et al*, 2004) The pooled analysis for these 11 studies, together with the study presented here, included 6528 breast cancer cases and provided an odds ratio (95% CI) of 1.07 (0.94, 1.22) (Figure 1

). The pooled estimates from differing sensitivity analyses to assess the effect of different exposure measures did not differ substantively from this estimate (all odds ratios for these analyses were between 1.05 and 1.08). There was heterogeneity between the studies (*P*=0.01), which was not explained by menopausal status (*P*=0.34). The Egger test did not suggest strong evidence of small study bias (*P*=0.23), although the Begg test provided more evidence of this (*P*=0.07). The funnel plot (Figure 2

) shows the influence of two small studies with large positive effects on the Begg test result, and examination of the Forrest plot (Figure 1) suggests that these two studies are an important source of heterogeneity. When these two studies were removed from the meta-analysis (leaving *N*=6165 cases), the pooled odds ratio (95% CI) was 1.03 (0.93, 1.14) with no strong evidence of heterogeneity between studies (*P*=0.23). Other than being two of the smallest and least precise studies, it can be seen from Table 3 that there is nothing that makes these two studies specifically different from all other studies.

## DISCUSSION

Our results suggest that there is no association between a woman smoking before the birth of her first child and breast cancer. Our finding that smoking around the time of puberty was not associated with increased risk of breast cancer is consistent with a number of other studies.(O'Connell *et al*, 1987; Rohan and Baron, 1989; Chu *et al*, 1990; Ewertz, 1990, 1993; Field *et al*, 1992; Smith *et al*, 1994; Baron *et al*, 1996; Band *et al*, 2002) Taken together, these findings suggest that smoking during the period of breast tissue development and before final cellular differentiation (ie before completion of a first pregnancy) is not associated with breast cancer.

### Study limitations

The majority of our cases were prevalent and survivor bias may be an important limitation. Breast cancer in the UK is associated with a survival rate of 70% over 5 years (Coleman *et al*, 1999). Our study, and other case–control studies of this association, may therefore exclude a number of women with aggressive disease. If smoking prior to first birth is strongly associated with survival, then we may have missed an important association. However, two prospective studies of incident cases only did not find an association (Egan *et al*, 2002; Reynolds *et al*, 2004), and when we restricted our analyses to incident cases only, although imprecise there was no evidence of an association.

Breast cancer cases were not confirmed histologically and self-reported breast cancer may be inaccurate. However, over 80% of the cases were identified from at least two sources, including medical records, cancer registers or self-report. Medical record and cancer register cases are likely to have been confirmed by histological reports and when sensitivity analyses were performed using breast cancer data from each of just one of the three sources (cancer register, general practice medical records, self-report) the results were unchanged. By combining information from all three sources, it is likely that most cases have been identified.

An important limitation of our, and other, studies is the small number of cases and hence imprecision of the results. This highlights the importance of pooling results from all studies in order to provide a precise estimate of the overall effect.

### Smoking after first birth

Although imprecise and not an *a priori* hypothesis, our results suggest that smoking solely after a woman's first birth may be protective against breast cancer. Two other studies have also found reduced risk of breast cancer associated with smoking only in the period after a first birth (Band *et al*, 2002; Lash and Aschengrau, 2002). Some compounds in cigarettes inhibit the aromatisation of androgens to oestrogens and enhance the formation of oestrodiol metabolites with low oestrogenic activity (Michnovicz *et al*, 1986; Kadohama *et al*, 1993). Hence it has been suggested that smoking in later life may be associated with reduced risk of breast cancer through mechanisms related to decreased oestrogen activity (Band *et al*, 2002). However, results in this area have been inconsistent with two studies finding increased risk of breast cancer among women who begin smoking only after the birth of their first child (Lash and Aschengrau, 1999; Kropp and Chang-Claude, 2002), and two prospective studies found no association (Egan *et al*, 2002; Reynolds *et al*, 2004).

## CONCLUSION

Smoking around the time of breast tissue development and before full differentiation – between puberty and prior to completion of the first pregnancy – is not associated with increased risk of breast cancer. Smoking is associated with a number of adverse health outcomes and young women should be discouraged from taking up smoking irrespective of any association or lack of association with breast cancer.
